# Talking to your car can drive you to distraction

**DOI:** 10.1186/s41235-016-0018-3

**Published:** 2016-11-14

**Authors:** David L. Strayer, Joel M. Cooper, Jonna Turrill, James R. Coleman, Rachel J. Hopman

**Affiliations:** University of Utah, Salt Lake City, UT USA

**Keywords:** Cognitive workload, Cognitive distraction, Driving, Driver distraction, Aging, Divided attention, Multitasking

## Abstract

This research examined the impact of in-vehicle information system (IVIS) interactions on the driver’s cognitive workload; 257 subjects participated in a weeklong evaluation of the IVIS interaction in one of ten different model-year 2015 automobiles. After an initial assessment of the cognitive workload associated with using the IVIS, participants took the vehicle home for 5 days and practiced using the system. At the end of the 5 days of practice, participants returned and the workload of these IVIS interactions was reassessed. The cognitive workload was found to be moderate to high, averaging 3.34 on a 5-point scale and ranged from 2.37 to 4.57. The workload was associated with the intuitiveness and complexity of the system and the time it took participants to complete the interaction. The workload experienced by older drivers was significantly greater than that experienced by younger drivers performing the same operations. Practice did not eliminate the interference from IVIS interactions. In fact, IVIS interactions that were difficult on the first day were still relatively difficult to perform after a week of practice. Finally, there were long-lasting residual costs after the IVIS interactions had terminated. The higher levels of workload should serve as a caution that these voice-based interactions can be cognitively demanding and ought not to be used indiscriminately while operating a motor vehicle.

## Significance

Motor vehicle crashes are the leading cause of accidental injury deaths in the US (National Safety Council White, 2010). Distractions of one kind or another have been observed in 66 % of cases within the 6 s preceding a crash (Carney, McGehee, Harland, Weiss, & Raby, 2015). Voice-activated features may seem to be a natural solution to driver distraction, allowing drivers to keep their eyes on the road and their hands on the steering wheel. In fact, many newer-model vehicles come equipped with voice-activated systems that allow drivers to adjust climate control, select music, place and receive phone calls, send and read textual messages, and interact with social media. The impact of these voice-based commands on the motorist’s cognitive workload is unknown; however, voice-command laboratory surrogates with perfect reliability have been shown to produce surprisingly high levels of workload (Strayer, Cooper, Turrill, Coleman, & Hopman, 2015a, Strayer et al., 2015b). Moreover, older adults are likely to have greater difficulty using these voice-based systems and, ironically, they are more likely to purchase new vehicles equipped with these features (Sivak, 2013). Finally, the effect of practice with these voice-command systems has not been fully explored and it is unknown if the higher levels of cognitive workload abate with extended practice. These issues take on greater significance given a large segment of the driving public may use these systems in the coming years.

## Background

In order to allow drivers to maintain their eyes on the forward roadway, nearly every vehicle sold in the US and Europe can now be optionally equipped with an in-vehicle information system (IVIS). Using voice commands, drivers can access functions as varied as voice dialing, music selection, GPS destination entry, and even climate control. Voice-activated features would seem to be a natural evolution in vehicle safety that requires little justification. However, a large and growing body of literature cautions that auditory/vocal tasks may have unintended consequences that adversely affect traffic safety (e.g., Bergen, Medeiros-Ward, Wheeler, Drews, & Strayer, 2013).

The National Highway Traffic Safety Administration (NHTSA) is in the process of developing voluntary guidelines to minimize driver distraction created by electronic devices in the vehicle. There are three planned phases to the NHTSA guidelines. The phase 1 guidelines, entered into the Federal Register on March 15, 2012, address visual–manual interfaces for devices installed by vehicle manufactures. The phase 2 guidelines, scheduled for release sometime in 2016, will address visual–manual interfaces for portable and aftermarket electronic devices. Phase 3 guidelines will address voice-based auditory interfaces for devices installed in vehicles and for portable aftermarket devices.

Currently, there are no unified regulations regarding the use of wireless technology in the vehicle—the NHTSA phase 1 guidelines are voluntary and it is unknown whether any of the currently available vehicles meet these guidelines. With the explosive growth in technology, the problem of driver distraction is poised to become much more acute.

### Benchmarking cognitive distraction

Our prior research provided a benchmark for the cognitive workload associated with common in-vehicle activities (Strayer et al., 2015a, 2015b; see also Cooper, Ingebretsen, & Strayer, 2014; and Strayer, Turrill, Coleman, Ortiz, & Cooper, 2014). In our studies, we developed and validated a cognitive distraction scale based on converging operations from the laboratory, driving simulator, and an instrumented vehicle driven in a residential section of Salt Lake City. Our research shows that the distraction potential can be reliably measured, that cognitive workload systematically varies as a function of the secondary task performed by the driver, and that some activities, particularly newer voice-based interactions in the vehicle, are associated with surprisingly high levels of mental workload.

We obtained workload ratings attributable to cognitive sources by comparing seven different concurrent tasks with a “single-task” condition where the drivers did not perform any concurrent secondary-task activity (Strayer et al., 2015b). The seven tasks were listening to the radio, listening to a book on tape, talking to a passenger, talking on a hands-free cell phone, talking on a hand-held cell phone, interacting with a simple voice messaging system, and a cognitively demanding Operation Span (OSPAN) task that was used for calibration.^1^ In our distraction scale, the non-distracted single-task driving anchored the low-end (category 1) and the mentally demanding OSPAN task anchored the high-end (category 5) of the scale. Using this method, we found that activities such as listening to the radio or an audio book were not very distracting. Other activities, such as conversing with a passenger or talking on a hand-held or hands-free cell phone, were associated with moderate increases in cognitive distraction. Finally, activities such as using a speech-to-text system to send and receive short text or e-mail messages produced a surprisingly high level of cognitive distraction.

The speech-to-text system that we evaluated in the laboratory is noteworthy because the speech-recognition portion of the system was perfectly reliable and there was no requirement to review, edit, or correct garbled translations. In our research protocol, perfect speech recognition was implemented using a “Wizard-of-Oz” paradigm (Kelley, 1983; Lee, Caven, Haake, & Brown, 2001), in which the participant’s speech was secretly entered into the computer by the experimenter with no transcription errors. Consequently, drivers did not need to take their eyes off the road or their hands off the steering wheel when making these voice-based interactions. Nevertheless, this “best case” speech-to-text e-mail/text message system received a category 3 rating on the cognitive distraction scale.

In our 2014 research (Strayer et al., 2014), we examined voice-based interactions in greater detail. We found that just listening to voice messages without the possibility of generating a reply was associated with a cognitive workload rating comparable to that of conversing on a cell phone (i.e., category 2). However, when drivers composed replies to these messages, the workload rating increased to category 3 on the cognitive distraction scale. Like our earlier testing, this laboratory-based system was perfectly reliable. We also found no systematic difference between the natural (i.e., human) and synthetic (i.e., computerized) delivery of the messages. This latter finding suggests that there is little to be gained by improving the quality of the synthetic speech, at least with regard to the driver’s mental workload.

Our 2014 research also evaluated Apple’s intelligent personal assistant, *Siri*, to send and receive text messages, update Facebook or Twitter, and to modify and review calendar appointments. To create a completely hands-free version of the interaction, a lapel microphone was clipped to the participant’s collar and they activated *Siri* with the command “*Hey Siri*” at which point a researcher manually activated the device. Drivers neither looked at nor made physical contact with the iPhone during these interactions. Even so, the workload ratings for these interactions exceeded category 4 on our workload scale. Moreover, there were two crashes in the driving simulator study when participants were using *Siri*.

The primary difference between our laboratory-based speech-to-text system and the *Siri*-based interactions was the reliability of the system (see also Strayer et al., 2015a). *Siri* was error-prone, producing different responses to seemingly identical commands. In other circumstances, *Siri* required exact phrasing to accomplish specific tasks and subtle deviations from that phrasing would result in failure. Moreover, when there was a failure to properly dictate a message, *Siri* required starting the interaction over since there was no way to modify/edit a message or command. For these reasons and others, voice-based interactions using an intelligent personal assistant such as *Siri* were significantly more mentally demanding than conversing on a cell phone.

### Research objectives and experimental overview

The current research addresses several important issues related to the assessment of cognitive workload in the vehicle. First, our prior research examined drivers who were in their mid-20s (e.g., the average age of participants in the Strayer et al. (2015b) study was 23). This younger cohort tends to be more tech-savvy than an older population: it is unclear how demanding older drivers will find these voice-based interactions. This issue gains importance because drivers between the ages of 55 and 64 years are the most likely to purchase new vehicles equipped with voice-command technology to control infotainment and other vehicle functions (Sivak, 2013). In fact, laboratory studies have documented substantially greater costs of multitasking for older adults (e.g., Hartley & Little, 1999; Kramer & Larish, 1996; McDowd & Shaw, 2000); therefore, it is likely that the workload scale developed in our prior research is a conservative estimate of the cognitive workload experienced by older drivers interacting with these voice-based systems.

Second, our prior research examined the driver’s cognitive workload soon after they had been introduced to the vehicle, with minimal training (i.e., 15 minutes or less) using the vehicle and the IVIS. The old adage “practice makes perfect” suggests that extended practice with the IVIS may reduce or even eliminate the interference caused by these voice-based interactions. For practice to be effective, however, the system needs to be intuitive and error free with a consistent mapping between input–output operations (e.g., Shiffrin & Schneider, 1977). Because many of the systems that are currently available tend to be complex and error prone, with inconsistent behavior (e.g., Cooper et al., 2014), there are limits on how much improvement can be expected with extended practice.

Our study recruited male and female drivers between the ages of 21 and 70 years to participate in a weeklong evaluation of IVIS interactions in one of ten different model-year 2015 automobiles. After familiarization with the vehicle, participants were trained on how to interact with the voice-based system to perform common IVIS tasks (e.g., dialing, radio tuning). Following this initial orientation, they were tested on the IVIS interactions using the method that we developed to assess cognitive workload in the vehicle (e.g., Strayer et al., 2015b). Participants then took the vehicle home for 5 days and practiced interacting with the IVIS. At the end of 5 days of practice, participants returned and were retested on the cognitive workload of these same IVIS interactions. This design allowed us to evaluate the effects of age and practice on IVIS interactions.

## Methods

### Participants

Following approval from the institutional review board (IRB), participants were recruited by word of mouth and flyers posted on the University of Utah campus. They were compensated $250 upon completion of the weeklong study. Data were collected from July 4th of 2014 through June 18th of 2015.

The study included 257 subjects (130 females, 127 males). Ages ranged from 21 to 70 years old (*x̄* = 44 years). Participants were recruited to provide a minimum of four male and four female licensed drivers in each of the three age groups, 21–34, 35–53, and 54–70 years, for each of the ten vehicles. An accounting of participants’ gender and age group is provided in Table 1.

**Table 1:** Distribution of age and gender for each of the vehicles used in the experiment

Vehicle model	21–34 years		35–53 years		54–70 years	
	Male	Female	Male	Female	Male	Female
Buick LaCrosse	4	4	4	4	5	4
Chevy Equinox	4	4	5	4	5	4
Chevy Malibu	4	4	5	5	4	5
Chrysler 200c	4	5	4	5	4	5
Ford Taurus	4	4	5	5	4	4
Hyundai Sonata	4	4	5	4	5	5
Mazda 6	4	4	4	4	4	5
Nissan Altima	4	5	4	4	4	4
Toyota 4Runner	4	4	4	4	4	4
VW Passat	4	5	4	4	4	4

Prior to participation in the research, the University of Utah’s Division of Risk Management ran a Motor Vehicles Record report on each prospective participant to ensure a clean driving history (e.g., no at-fault accidents in the past five years) and eligibility to be registered as a University driver. In addition, following University of Utah policy, each participant was required to complete a 20-minute online defensive driving course and pass the certification test. Participants reported between 5 and 55 years of driving experience (*x̄* = 28 years). Additionally, participants reported driving an average of 160 miles per week. All participants were recruited from the greater Salt Lake area and spoke well-articulated English.

### Materials and equipment

Ten 2015 model-year vehicles equipped with automatic transmissions were used in this research (see Table 1 for a complete breakdown of the different vehicles used in the study). In each vehicle, voice-based interactions with the IVIS were initiated with the press of a button located on the steering wheel and ended either automatically or with a second press of the button, depending on the vehicle and function. Each of the ten vehicle systems allowed drivers to complete contact calling and number dialing tasks through a Bluetooth-paired cellphone.

Dual-Vision XC cameras, manufactured by Rosco Vision Systems, were installed in the vehicles by a qualified technician. Cameras were mounted under the rear view mirror, providing a view of the forward roadway and of the driver’s face. An infrared illuminator was installed in each vehicle for nighttime video recording. The cameras also included an embedded GPS system. Cameras were set to automatically begin recording audio, video, and GPS data as soon as the vehicle ignition was turned on by the driver and to stop recording when the vehicle ignition was turned off. Video data were recorded at 3.5 frames per second at standard VGA resolution.

During the first day of the study (session 1) and on the last day of the study (session 2), participants wore a head-mounted Detection Response Task (DRT) device that consisted of an LED light mounted to a flexible arm that was connected to a headband, a micro-switch attached to the participant’s left or right thumb (the switch was attached to the hand opposite that of the vehicle’s steering wheel voice-activation button), and a dedicated microprocessor to handle all stimulus timing and response data. The light was positioned in the periphery of the participant’s left eye (approximately 15° to the left and 7.5° above the participant’s left eye) so that it could be seen while looking at the forward roadway but did not obstruct the view of the driving environment. The stimulus presentation configuration adhered to the International Standards Organization (ISO) standard 17488 with red LED stimuli configured to flash every 3–5 s. Data were collected using an Asus Transformer Book T100s with quad-core Intel® Atom™ processors running at 1.33 GHz.

An auditory version of the OSPAN task, developed by Watson and Strayer (2010), was used to induce a high workload baseline during testing. This task required participants to recall single syllable words in serial order while solving mathematical problems. In the auditory OSPAN task, participants were asked to remember a series of two to five words that were interspersed with math-verification problems (e.g., given “[3/1] − 1 = 2?”—“cat”—“[2 × 2] + 1 = 4?”—“box”—RECALL, the participant should have answered “true” and “false” to the math problems when they were presented and recalled “cat” and “box” in the order in which they were presented when given the recall probe). In order to standardize presentation for all participants, a prerecorded version of the task was created and played back during testing.

Subjective workload ratings were collected using the NASA TLX survey developed by Hart and Staveland (1988). After completing each of the conditions (single-task, IVIS, and OSPAN; see the “Procedure” section below for details) in the experiment, participants responded to the NASA TLX survey consisting of six questions that used a 21-point Likert scale, ranging from “very low” to “very high”. The questions in the NASA TLX were:

1. How mentally demanding was the task?
2. How physically demanding was the task?
3. How hurried or rushed was the pace of the task?
4. How successful were you in accomplishing what you were asked to do?
5. How hard did you have to work to accomplish your level of performance?
6. How insecure, discouraged, irritated, stressed, and annoyed were you?

A study facilitator was assigned to each participant for the duration of the data collection session. Facilitators were trained to precisely administer the research procedure and adhered to a scripted evaluation protocol. Additionally, facilitators were responsible for ensuring the safety of the driver, providing in-car training, and delivering task cues to participants.

### Procedure

Before the study began, participants filled out an IRB approved consent form and a brief intake questionnaire to assess basic characteristics of phone and driving usage and experience. Participants were then familiarized with the controls of the instrumented vehicle, adjusted the mirrors and seat, and were informed of the tasks that would be completed while driving. The first portion of training involved an introduction to the DRT device. Participants were fitted with the device and were instructed on its functionality. Once comfortable with the general procedure, they were allowed to practice with the DRT device until they felt comfortable with its usage. In most cases, participants were comfortable with the functionality of the device within a couple of minutes. Participants were provided training on the functionality of the IVIS system and asked to complete a series of contact calling, number dialing, and radio tuning tasks until they reached proficiency. Participants then completed a three-minute orientation for each of the tasks in the IVIS condition and a three-minute orientation of the OSPAN task while the vehicle was parked. A practice loop within a parking lot was completed in order to familiarize the participant with the handling of the vehicle.

Next, participants completed one circuit around the 2.7 mile driving loop, located in the Avenues section of Salt Lake City, UT, in order to become familiar with the route itself. The route provided a suburban/residential driving environment and contained seven all-way controlled stop signs, one two-way stop sign, and two stoplights. Given the restricted usage characteristics of the roadway, traffic remained relatively consistent during testing. After the practice drive, participants began the experimental portion of the study. In total, participants drove the vehicle for approximately 20 minutes before the initial data collection began.

Six tasks were given to participants during the IVIS condition of the study; each involved the use of the vehicle’s unique voice-activated infotainment system. The tasks were initiated once participants reached pre-specified locations that were chosen to allow participants approximately 1.5 minutes to complete each task. If the participant was unable to complete a task before the next task was to begin, they were told to abandon that first task and move on to the new one.

All of the tasks in the IVIS condition began when participants pressed the voice activation button located on the steering wheel. Once initiated, each of the tasks was completed through auditory plus vocal system interactions. System interactions were performed in a fixed order and alternated between completing a phone calling task and a radio-tuning task. The tasks in the IVIS condition were as follows:

- Task 1: “Call from your contacts Joel Cooper”
- Task 2: “Tune your radio to 98.3 FM”; once completed, “Tune your radio to 1320 AM”
  - Task 2b (for the Nissan and Volkswagen vehicles): “Call from your contacts Chris Hunter”
- Task 3: “Dial your own phone number”
- Task 4: “Tune your radio to 1160 AM”; once completed, “Tune your radio to 90.1 FM”
  - Task 4b (for the Nissan and Volkswagen vehicles): “Dial your own phone number”
- Task 5: “Call from your contacts Amy Smith at work”
- Task 6: “Dial your own phone number”

Participants were then familiarized with the specific requirements of the upcoming condition and were told that their task was to follow the route previously practiced while complying with all local traffic rules, including obeying a 25 miles per hour (mph) speed limit. Throughout each of the three experimental conditions (single-task, IVIS, and OSPAN), the driver performed the DRT task. Each of the conditions required the driver to complete one loop of the 2.7-mile course and the order of the conditions was counterbalanced across participants. The OSPAN condition induced a continuous secondary-task load, whereas the task load in the IVIS condition was intermittent. Any driving sections with turns were excluded from the DRT and video analyses to minimize the potential of a manual confound.

At the conclusion of the first day of testing (session 1), participants were given a logbook to document their interactions with the IVIS during the ensuing 5 days. Participants were encouraged to practice using the IVIS system on their own time with special emphasis given to contact calling, number dialing, and radio station selection. Once familiar with the journaling and instructions for the week, participants took the research vehicle home and began the practice portion of the study. Following the five-day practice interval, participants returned on the last day for evaluation (session 2). The data collection protocol for session 2 was identical to that of session 1 except that the extensive IVIS training was no longer necessary.

### Design

The core experimental design was a 3 (Age) × 10 (Vehicle) × 3 (Condition) × 2 (Session) split-plot factorial. Age was a between-subject factor and included three age groups, 21–34, 35–53, and 54–70 years.^2^ Vehicle was also a between-subject factor and included ten 2015 model-year vehicles: a Buick LaCrosse with IntelliLink, a Chevy Equinox with MyLink, a Chevy Malibu with MyLink, a Chrysler 200c with Uconnect, a Ford Taurus with Sync MyFord Touch, a Hyundai Sonata with Blue Link, a Mazda 6 with Connect, a Nissan Altima with NissanConnect, a Toyota 4Runner with Entune, and a Volkswagen Passat with Car-Net. Condition was a three-level within-subject factor (single-task, IVIS, and OSPAN conditions). Session was also a within-subject factor and refers to the first day of testing (session 1) and the last day of testing (session 2) that were separated by 5 days of practice with the IVIS system. The three conditions in each session were performed in a counterbalanced order across participants. Interactions with the IVIS involved two number dialing tasks, two contact calling tasks, and four radio tuning tasks, with the exception that participants driving the Nissan and Volkswagen vehicles completed three number dialing tasks and three contact calling tasks because these vehicles did not support vocal radio tuning. Additionally, because the DRT analysis allowed for a differentiation between on-task performance (i.e., the time when participants were actively engaged in the IVIS interactions) and off-task performance (i.e., the period of time between IVIS tasks when the driver was not interacting with the IVIS but rather was driving as in the single-task condition), Condition had four factors (i.e., single-task, IVIS off-task, IVIS on-task, and OSPAN) when assessing the effects of IVIS interactions on DRT performance.

### Dependent measures

Cognitive workload was determined by a number of performance measures. These measures were derived from the DRT task, subjective reports, and analysis of video recorded during the experiment.

DRT data were cleaned following procedures specified in ISO 17488 (2015). Consistent with the standard, all responses briefer than 100 ms or greater than 2500 ms were rejected for calculations of reaction time (RT). Responses that occurred later than 2500 ms from the stimulus onset were coded as misses. Any DRT data collected during turns was flagged and removed from analysis. During testing of the IVIS interactions, trial engagement was flagged by the facilitator through a keyboard press which allowed the identification of segments of the IVIS condition when the participant was actively engaged in an activity (IVIS-1) or had finished that activity and was operating the vehicle without voice-based interactions (IVIS-0).

- DRT MANOVA. An overall analysis that statistically combined the effects of RT and hit rate (see the “Hit rate” section below).
- DRT RT. Defined as the sum of all valid RTs to the DRT task divided by the number of valid RTs.
- DRT hit rate. Defined as the number of valid responses divided by the total number of stimuli presented during each condition.
- DRT residual costs. To evaluate the residual effects of secondary task interactions on DRT RT, performance in the off-task segments of the drive was sorted into 3-s bins relative to the time that the off-task interval began. For example, a DRT event occurring 5 s after the end of an IVIS interaction would be sorted into the second bin.

Following each drive, participants were asked to fill out a brief questionnaire that posed eight questions related to the just-completed task. The first six of these questions were from the NASA TLX; the final two assessed the intuitiveness and complexity of the IVIS interactions.

- Subjective: NASA TLX. Defined as the response on a 21-point scale for each of the six subscales of the TLX (Mental, Physical, Temporal, Performance, Effort, and Frustration).
- Subjective: Intuitiveness and complexity. Defined as the response on a 21-point scale to questions on task intuitiveness (i.e., “how intuitive, usable, and easy was it to use the system”) and complexity (i.e., “how complex, difficult, and confusing was it to use the system”).

Task completion time, glance location, and practice frequency were derived from the video recordings. Task completion time and glance location were available for 214/257 participants, while video analysis of practice frequency was available for 180/257 participants. In all cases, frame-by-frame analysis was completed, sampling two frames per second. The reliability of the coding was assessed through an evaluation of the time-on-task data from the DRT and the coded videos. Results from this assessment indicated that the two sources showed a nearly identical pattern (*r* = 0.96).

- Video: Task completion time. Task completion time was defined as the time from the moment participants first pressed the voice activation button to the time that the same button was pressed to terminate a task, or in the case of radio tuning, the moment when the system accurately carried out the requested task. Task completion time reflects the average task duration across the six tasks in the IVIS condition.
- Video: Glance location. Defined as the percentage of all visual glances that fell within the forward roadway, the dashboard region, or the right, left, and rear-view mirrors.
- Video: Practice frequency. Defined as the count of IVIS voice interactions during the 5-day practice session where participants practiced using the voice assistant to call a contact, dial a number, tune the radio, or engage in other voice tasks.

## Results

### Detection Response Task

**Fig. 1:**
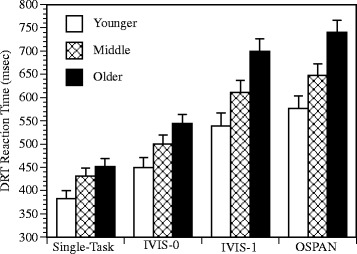
Mean DRT reaction time (in milliseconds) for the single-task, IVIS-0 (“off-task”), IVIS-1 (“on-task”), and OSPAN conditions. The data are plotted for younger, middle, and older age groups. *Error bars* reflect the 95 % confidence interval around the point estimate

**Fig. 2:**
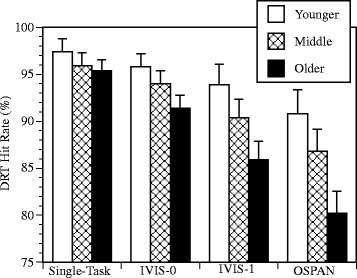
Mean DRT hit rate (an accuracy measure expressed as a percentage and computed by determining the number of valid responses divided by the total number of responses) for the single-task, IVIS-0 (“off-task”), IVIS-1 (“on-task”), and OSPAN conditions. The data are plotted for younger, middle, and older age groups. *Error bars* reflect the 95 % confidence interval around the point estimate

**Fig. 3:**
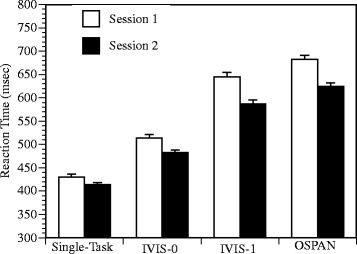
Mean DRT reaction time (in milliseconds) for the single-task, IVIS-0 (“off-task”), IVIS-1 (“on-task”), and OSPAN conditions. The data are plotted for the first testing day (session 1) and the last testing day (session 2). *Error bars* reflect the 95 % confidence interval around the point estimate

**Fig. 4:**
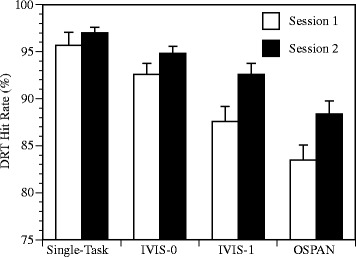
Mean DRT hit rate (an accuracy measure expressed as a percentage and computed by determining the number of valid responses divided by the total number of responses) for the single-task, IVIS-0 (“off-task”), IVIS-1 (“on-task”), and OSPAN conditions. The data are plotted for the first testing day (session 1) and the last testing day (session 2). *Error bars* reflect the 95 % confidence interval around the point estimate

The DRT data reflect the response to the onset of the red light in the peripheral detection task. RT was measured to the nearest millisecond. Hit rate was calculated based on a response to the red light, which was coded as a “hit”, and a non-response to a red light, which was coded as a “miss”. The RT and hit rate data for the DRT task are plotted as a function of Age × Condition in Figs. 1 and 2, respectively. The data from the DRT task are also plotted as a function of Session × Condition in Figs. 3 and 4, respectively. The data are broken down by active involvement in the IVIS condition, denoted by a suffix of “-1” (i.e., IVIS-1) or when participants were operating the vehicle without concurrent secondary-task interaction, denoted by a suffix of “-0” (i.e., IVIS-0).

The DRT is inversely related to the workload in the driving task (e.g., Strayer et al., 2015a, 2015b). Thus, increases in RT and decreases in hit rate are indicative of an increase in the workload experienced by the driver. As can be seen in Figs. 1 and 2, RT increased and hit rates decreased as a function of the experimental condition and the age of the participant. Additionally, the age-related differences observed in the single-task baseline were amplified in the IVIS-1 condition. Perusal of Figs. 3 and 4 shows that RT decreased and hit rates increased with practice and the practice effects observed in the single-task baseline were exacerbated in the IVIS-1 condition.

#### MANOVA

The DRT data were first analyzed using a 3 (Age) × 10 (Vehicle)^3^ × 4 (Condition) × 2 (Session) MANOVA that included both reaction time and hit rate as dependent variables.^4^ There were significant main effects of Age (*F*(4, 454) = 14.07, *p* < 0.001, η^2^ = 0.110), Condition (*F*(6, 1362) = 164.86, *p* < 0.001, η^2^ = 0.421), and Session (*F*(2, 226) = 48.61, *p* < 0.001, η^2^ = 0.301). In addition, Condition interacted with Age (*F*(12, 1362) = 8.15, *p* < 0.001, η^2^ = 0.067), Vehicle (*F*(54, 1362) = 1.53, *p* = 0.009, η^2^ = 0.057), and Session (*F*(6, 1362) = 12.54, *p* < 0.001, η^2^ = 0.052). None of the other effects were significant.

#### Reaction time

The reaction time data from the DRT were analyzed using a 3 (Age) × 10 (Vehicle) × 4 (Condition) × 2 (Session) Analysis of Variance (ANOVA). The analysis revealed significant main effects of Age (*F*(2, 227) = 31.71, *p* < 0.001, η^2^ = 0.218), Condition (*F*(3, 681) = 894.29, *p* < 0.001, η^2^ = 0.798), and Session (*F*(1, 227) = 84.65, *p* < 0.001, η^2^ = 0.272). In addition, as can be seen in Fig. 1, Condition interacted with Age (*F*(6, 681) = 15.75, *p* < 0.001, η^2^ = 0.122), Vehicle (*F*(27, 681) = 2.00, *p* = 0.002, η^2^ = 0.074), and Session (*F*(3, 681) = 16.62, *p* < 0.001, η^2^ = 0.068). None of the other effects were significant.

#### Hit rate

The hit rate data from the DRT task were analyzed using a 3 (Age) × 10 (Vehicle) × 4 (Condition) × 2 (Session) ANOVA. The analysis revealed significant main effects of Age (*F*(2, 227) = 17.87, *p* < 0.001, η^2^ = 0.136), Condition (*F*(3, 681) = 129.15, *p* < 0.001, η^2^ = 0.363), and Session (*F*(1, 227) = 53.61, *p* < 0.001, η^2^ = 0.191). In addition, as shown in Fig. 2, Condition interacted with Age (*F*(6, 681) = 7.94, *p* < 0.001, η^2^ = 0.065), Vehicle (*F*(27, 681) = 1.87, *p* = 0.005, η^2^ = 0.069), and Session (*F*(3, 681) = 12.44, *p* < 0.001, η^2^ = 0.052). None of the other effects were significant.

The Condition × Age interaction (Figs. 1 and 2) indicates that the costs of the IVIS interactions were greater for older adults than for younger adults. RT increased with age by 18.2 % in the single-task condition and by 29.7 % in the IVIS-1 condition. A similar analysis of hit rates found a decrease with age of 2.1 % in the single-task condition and of 8.5 % in the IVIS-1 condition. This interaction was also found in the log transformed RT data (*F*(2, 227) = 10.17, *p* < 0.001, η^2^ = 0.071).^5^

The Condition × Session interaction (Figs. 3 and 4) indicates that the effects of practice were more pronounced when participants were using the IVIS than when they were in the single-task condition. RT decreased with practice by 3.5 % in the single-task condition and by 9.0 % in the IVIS-1 condition. A similar comparison on hit rates found an increase with practice of 1.4 % in the single-task condition and of 5.7 % in the IVIS-1 condition.

The MANOVA reported above found a significant Condition × Vehicle interaction that requires additional analyses for clarity in the interpretation. The interaction could be due to difficulties operating the vehicle, workload differences with the IVIS interactions, or a combination of the two. To discriminate between these interpretations, we created a composite of the DRT measures obtained from the IVIS-1 condition by taking the weighted average of the z-transformed RT and hit rate data. This transformation was necessary because RT and hit rate are on different scales and the result was a score that was centered at 0 and the standard deviation was 1.^6^ A similar procedure was used to compute the single-task and OSPAN composite scores.

**Fig. 5:**
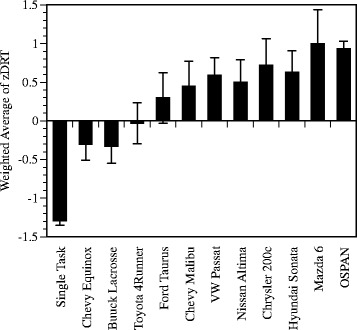
Weighted average of the z-transformed DRT data (i.e., DRT reaction time and hit rate) plotted as a function of Vehicle in the IVIS condition. *Error bars* reflect the 95 % confidence interval around the point estimate

Figure 5 presents the average of z-transformed DRT data plotted as a function of Vehicle in the IVIS-1 condition. For comparison, performance in the z-transformed DRT data for the single-task and OSPAN conditions is also included in Fig. 5. To better understand the Condition × Vehicle interactions reported above, a between-subject ANOVA was performed on the z-transformed data from the IVIS-1 condition. This analysis revealed a significant effect of Vehicle (*F*(9, 247) = 2.03, *p* = 0.037). By contrast, a similar analysis on the z-transformed data from the single-task and OSPAN conditions failed to yield a significant effect of Vehicle (*F*(9, 247) = 0.16, *p* = 0.320 and *F*(9, 247) = 1.04, *p* = 0.411, respectively). Moreover, an Analysis of Covariance (ANCOVA) on the data obtained in the IVIS-1 condition that held constant any performance differences in the single-task condition, found a significant effect of the IVIS voice-based interaction (*F*(9, 246) = 3.29, *p* < 0.001, η^2^ = 0.107). This pattern is important because it indicates that there were significant differences in DRT performance when participants were interacting with the IVIS, but there were no significant differences in DRT performance when they were just driving the vehicle. That is, the workload differences were associated with the IVIS voice-based interaction and not driving the vehicle by itself.

#### Residual costs

**Fig. 6:**
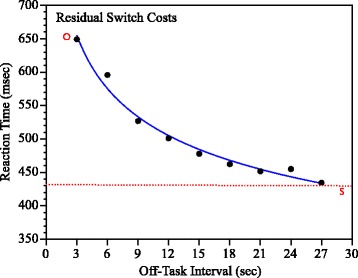
Residual switch costs in transitioning from on-task to off-task performance. The red “*O*” indicates average OSPAN RT from the DRT task. The red “*S*” indicates the average single-task RT from the DRT task. Off-task performance is distributed into 3-s intervals (relative to when the on-task activity terminated). The filled circles reflect the average RT as a function of sorting bin and the *blue line* represents the best fitting power function describing the relationship between RT and bin, i.e., relating transition from on-task to single-task levels of performance. The *dotted red line* represents the critical t-value for significant differences from the single-task condition. Residual switch costs were significantly different from the single-task baseline up to 27 s after the on-task interval had terminated

A surprising finding was that the off-task performance in the DRT task differed significantly from single-task performance. Given that drivers were not engaged in any secondary-task activities during the off-task portions of the drive, it suggests that there were residual costs that persisted after the IVIS interaction had terminated. Figure 6 presents the residual costs plotted as a function of the time since the IVIS interaction terminated and the solid blue line reflects the best-fitting power function :

1 $$ f(x)=a*\left({x}^{-.1878}\right) $$

where *a* = exp(6.6915), with *R* ^*2*^ = 0.98.

The residual costs took a significant amount of time to dissipate. In fact, the data indicate that off-task performance reflects a mixture of “single-task” performance and the persistent costs associated with the IVIS interactions from the immediately preceding on-task period. One way to contextualize these residual costs is to use logic underlying the workload scale developed by Strayer et al. (2015b) to estimate, based solely on the DRT RT data, when the cognitive workload would reach a category 4 level (approximately 6 s), when it would reach a category 3 level (approximately 9 s), and when it would reach a category 2 level (approximately 15 s). The residual costs are notable because of their magnitude, their duration, and the fact that they are obtained even when there is no active switch to perform another task. They appear to reflect the lingering act of disengaging from the cognitive processing associated with the IVIS task and fully re-engaging attention to the driving environment. From a practical perspective, the data indicate that just because a driver terminates a voice-based interaction does not mean that they are no longer impaired. Indeed, the residual costs are at a category 3 level of impairment 9 s after the IVIS interaction had terminated. At the 25 mph speed limit in our study, drivers would have traveled over the length of a football field during this 9-s interval.

### Subjective

**Fig. 7:**
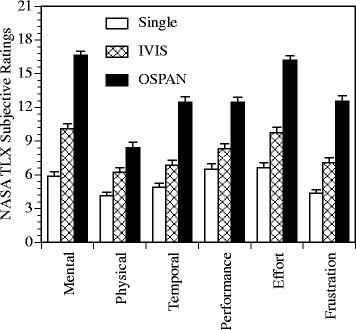
Mean NASA TLX ratings for the six sub-scales in the single-task, IVIS, and OSPAN conditions. *Error bars* reflect the 95 % confidence interval around the point estimate. A rating of 0 reflects a very low level of workload and a rating of 21 reflects a very high level of workload

**Fig. 8:**
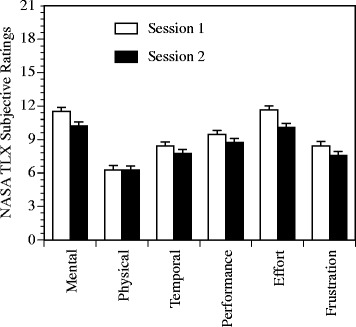
Mean NASA TLX ratings for the six sub-scales for the first testing day (*Session 1*) and the last testing day (*Session 2*). *Error bars* reflect the 95 % confidence interval around the point estimate. A rating of 0 reflects a very low level of workload and a rating of 21 reflects a very high level of workload

**Fig. 9:**
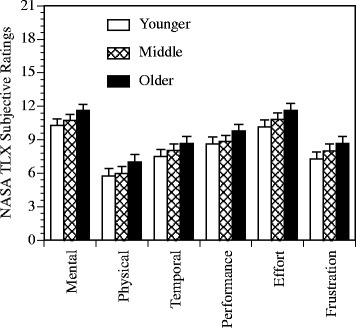
Mean NASA TLX ratings for the six sub-scales in the younger, middle, and older age groups. *Error bars* reflect the 95 % confidence interval around the point estimate. A rating of 0 reflects a very low level of workload and a rating of 21 reflects a very high level of workload

Subjective assessments of workload were made using the NASA TLX and supplementary questions on the intuitiveness and complexity of the IVIS systems. The NASA TLX is a subjective measure of workload that is composed of six sub-scales that range from 0 (no workload) to 21 (very high workload). As illustrated in Fig. 7, the subjective workload increased as a function of Condition. Figure 8 shows that the subjective workload decreased with practice. Figure 9 documents an increase in the subjective workload as a function of age of the participant.

#### NASA TLX

The six sub-scales of the NASA TLX were analyzed using a 3 (Age) × 10 (Vehicle) × 3 (Condition) × 2 (Session) ANOVA. There were significant main effects of Vehicle (*F*(54, 1362) = 1.47, *p* = 0.016, η^2^ = 0.055), Condition (*F*(12, 900) = 72.10, *p* < 0.001, η^2^ = 0.490), and Session (*F*(6, 222) = 28.51, *p* < 0.001, η^2^ = 0.435). In addition, Condition interacted with Age (*F*(24, 1880) = 2.46, *p* < 0.001, η^2^ = 0.032), Vehicle (*F*(108, 2724) = 1.60, *p* < 0.001, η^2^ = 0.060), and Session (*F*(12, 900) = 3.36, *p* < 0.001, η^2^ = 0.043). The Session × Vehicle (*F*(54, 1362) = 1.36, *p* = 0.045, η^2^ = 0.051) and the Session × Age × Vehicle interactions were also significant (*F*(108, 1362) = 1.30, *p* = 0.025, η^2^ = 0.094). None of the other effects were significant.

As with the DRT analysis described above, there was a significant Condition × Vehicle interaction in the TLX data, which is an analysis with parallel structure to the DRT. We created a composite of the TLX measures obtained from the IVIS condition by taking the weighted average of the z-transformed sub-scales of the TLX. A similar transform was used to compute the single-task and OSPAN composite scores. As with the DRT analysis, a main effect of the Vehicle holding constant any differences in single-task workload ratings (using ANCOVA) would indicate that subjective workload of the IVIS interactions differed as a function of Vehicle.

**Fig. 10:**
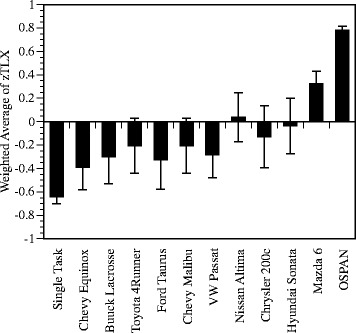
Weighted average of the z-transformed TLX data plotted as a function of Vehicle in the IVIS condition. *Error bars* reflect the 95 % confidence interval around the point estimate

Figure 10 presents the average of z-transformed TLX data plotted as a function of Vehicle in the IVIS condition. For comparison, performance in the single-task and OSPAN conditions is also included in Fig. 10. A between-subject ANOVA that compared the z-transformed data from the IVIS condition found a significant effect of Vehicle (*F*(9, 247) = 3.08, *p* = 0.002). A similar analysis on the z-transformed data found a significant effect of Vehicle in the single-task condition (*F*(9, 247) = 1.96, *p* = 0.044; a post-hoc analysis found that the Mazda, Hyundai, and Nissan vehicles had higher NASA TLX workload ratings than the VW and Equinox) but not in the OSPAN condition (*F*(9, 247) = 1.21, *p* = 0.292). An ANCOVA on the data from the IVIS condition that held constant the performance differences observed in the single-task condition also found a significant effect of IVIS interaction (*F*(9, 246) = 2.93, *p* = 0.003, η^2^ = 0.097). As with the DRT data reported above, this pattern is important because it indicates that there were significant differences in TLX performance when participants were interacting with the IVIS, over and above any differences of just driving the different vehicles. That is, the workload differences were associated with the IVIS voice-based interaction over and above any differences associated with operating the vehicle by itself.

#### Intuitiveness

**Fig. 11:**
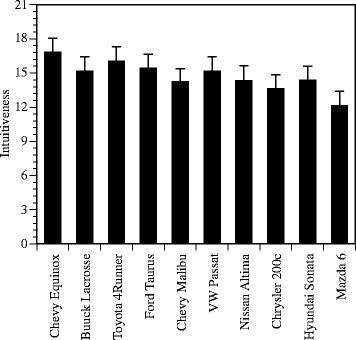
Mean ratings of intuitiveness (i.e., “how intuitive, usable, and easy was it to use the system”) for the different IVIS systems on a 21-point scale where 1 reflects “not at all” and 21 reflects “very much”. *Error bars* reflect the 95 % confidence interval around the point estimate

Participants were also asked to rate how intuitive, usable, and easy it was to use the IVIS. Figure 11 presents the intuitiveness ratings for the IVIS voice-based interactions on a 21-point scale where 1 reflects “not at all” and 21 reflects “very much”. A 3 (Age) × 10 (Vehicle) × 2 (Session) split-plot ANOVA found that intuitiveness varied as a function of Vehicle (*F*(9, 227) = 4.55, *p* < 0.001, η^2^ = 0.153). None of the other effects were significant (all other *p* values >0.14).

#### Complexity

**Fig. 12:**
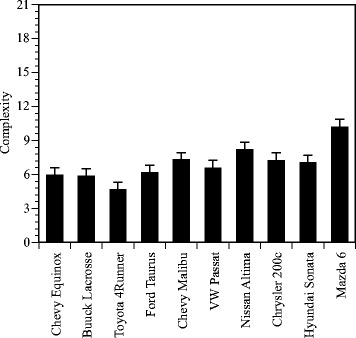
Mean ratings of complexity (i.e., “how complex, difficult, and confusing was it to use the system”) for the different IVIS systems on a 21-point scale where 1 reflects “not at all” and 21 reflects “very much”. *Error bars* reflect the 95 % confidence interval around the point estimate

Participants were also asked to rate how complex, difficult, and confusing it was to use the IVIS. Figure 12 presents the complexity ratings for the IVIS voice-based interactions on a 21-point scale where 1 reflects “not at all” and 21 reflects “very much”. A 3 (Age) × 10 (Vehicle) × 2 (Session) split-plot ANOVA found that complexity ratings varied as a function of Age (i.e., older adults found the IVIS interactions to be more complex; *F*(2, 227) = 6.21, *p* = 0.002, η^2^ = 0.052) and Vehicle (*F*(9, 227) = 4.82, *p* < 0.001, η^2^ = 0.160). None of the other effects was significant (all other *p* values >0.07).

### Video analysis

Three performance measures were derived from analysis of the video. These were task completion time, glance location, and practice frequency.

#### Task completion time

**Fig. 13:**
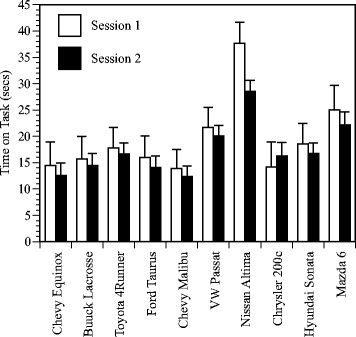
Mean time to complete the IVIS interactions for each vehicle. The data are plotted for the first testing day (*Session 1*) and the last testing day (*Session 2*). *Error bars* reflect the 95 % confidence interval around the point estimate

Task completion time, the average task duration for the six tasks in the IVIS condition, is plotted in Fig. 13. The data were analyzed using a mixed model ANOVA with Age and Vehicle as between-subject factors and Session as a within-subject factor. As can be seen in the figure, the time to complete the task varied as a function of Vehicle (*F*(9, 174) = 20.16, *p* < 0.001, η^2^ = 0.511). Additionally, there was a main effect of Session (*F*(1, 174) = 11.8, *p* < 0.001, η^2^ = 0.063) and the Vehicle × Session interaction was also significant (*F*(9, 174) = 2.04, *p* < 0.05, η^2^ = 0.095). However, the main effect of Age was not significant (*F*(2, 174) = 1.26, *p* = 0.285, η^2^ = 0.014) and neither were any of the interactions with Age. These data suggest that practice reduced task completion time but that the effect of this improvement was dependent on the vehicle. Not surprisingly given the long time on task, participants in the Nissan showed the greatest improvement in task completion time, moving from 37.6 s on average during the first session to 28.5 s during the final session; however, even after practice the duration of the interactions with the Nissan were longer than any of the other vehicles in the first session or practice.

#### Glance location

**Fig. 14:**
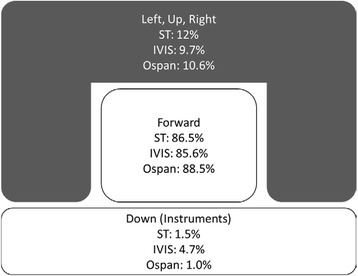
The distribution of glances to the forward roadway, instruments, and mirrors, broken down by single-task (*ST*), IVIS, and OSPAN conditions

The percentage of time that drivers spent looking forward, down, and scanning mirrors was analyzed using a 3 (Age) × 10 (Vehicle) × 3 (Condition) × 2 (Session) × 3 (Glance location) mixed model ANOVA with Age and Vehicle as between-subject factors and Session and Condition as within-subject factors. Glance location is plotted as a function of Condition in Fig. 14. There was a significant main effect of Glance location (*F*(2, 412) = 1247, *p* < 0.001, η^2^ = 0.868) and the Glance location × Condition interaction was also significant (*F*(4, 824) = 10.81, *p* < 0.001, η^2^ = 057). None of the other effects were significant.

A simplified 3 (Glance location) × 3 (Condition) repeated measures ANOVA was conducted on the data presented in Fig. 14. Both the main effect of Glance location (*F*(2, 856) = 126.17, p < 0.001, η^2^ = 0.983) and the Glance location × Condition interaction were significant (*F*(4, 856) = 52.9, *p* < 0.001, η^2^ = 0.198). Performing the voice tasks with the IVIS led to a reduction in the glance time to the mirrors and forward roadway with a corresponding increase in glance time to the dashboard displays. Similarly, performing the OSPAN task led to a reduction in the glance time to mirrors and dashboard displays with a corresponding increase in glance time to the forward roadway. Given that the primary task was to drive the vehicle and that the secondary tasks were primarily cognitive in nature, it is not surprising that drivers maintained their eyes on the forward roadway the majority of the time.

#### Practice frequency

**Fig. 15:**
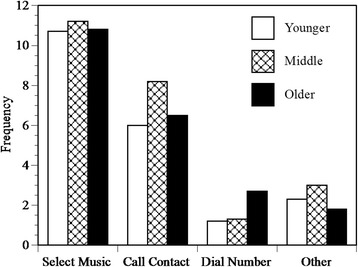
The mean number of interactions observed during the 5 days of practice. The data are plotted for younger, middle, and older age groups

The frequency of practice was coded from the video recordings. On average, participants completed a total of 21.8 (standard deviation = 19.3) voice-based tasks during the 5 days that they had the vehicle. As shown in Fig. 15, the age of the participant did not affect the amount of practice with the IVIS voice systems. Participants gained the most practice with the music selection task, followed by the contact-calling task, then the number dialing task. The practice data were analyzed using a 3 (Age) × 4 (Practiced item: contact call, number dial, music selection, other) ANOVA. The main effect of Practiced item was significant (*F*(3, 522) = 41.1, *p* < 0.001), but neither the main effect of Age nor the Age × Practiced item interaction were significant.

### The cognitive distraction scale

A primary objective of the current research was to compare the cognitive workload associated with IVIS interactions in ten different vehicles as drivers of different ages completed common IVIS voice-based tasks (e.g., voice dialing, music selection, etc.). Because the different dependent measures collected in this research were recorded on different scales, each was transformed to a standardized score. This involved z-transforming the two DRT measures and the six NASA TLX measures to have a mean of 0 and a standard deviation of 1. The standardized scores were then weighted and summed to provide an aggregate measure of cognitive distraction. Weighting was equally assigned to the DRT and TLX so that each accounted for 50 % of the collective rating. Finally, the aggregated standardized scores were scaled such that the non-distracted single-task driving condition anchored the low-end (category 1) and the OSPAN task anchored the high-end (category 5) of the cognitive distraction scale. For each of the other tasks, the relative position compared to the low and high anchors provided an index of the cognitive workload for that activity when concurrently performed while operating a motor vehicle. The four-step protocol for developing the cognitive distraction scale is listed below.

- Step 1: For each dependent measure, the standardized scores were computed using z_i_ = (x_i_ − X)/SD, where X refers to the overall mean and SD refers to the pooled standard deviation.
- Step 2: For each dependent measure, the standardized condition averages were computed by collapsing across subjects.
- Step 3: The standardized averages were computed with an equal weighting for secondary (i.e., DRT performance) and subjective (i.e., NASA TLX performance) metrics. The measures within each metric were also equally weighted. For example, the secondary task workload metric was comprised of an equal weighting of the measures DRT RT and DRT hit rate.
- Step 4: The standardized mean differences were range-corrected so that the non-distracted single-task condition had a rating of 1.0 and the OSPAN task had a rating of 5.0

5 $$ {\mathrm{X}}_{\mathrm{i}}=\left(\left(\left({\mathrm{X}}_{\mathrm{i}}- min\right)/\left( max- min\right)\right)*4.0\right)+1 $$

**Fig. 16:**
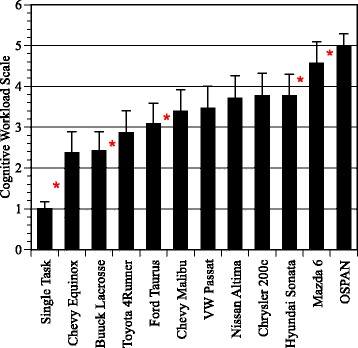
The cognitive workload scale for the IVIS interactions compared to single-task (category 1) and OSPAN (category 5). *Error bars* reflect the 95 % confidence interval around the point estimate. A red asterisk reflects adjacent pair-wise differences that were significant (*p* < 0.05)

The cognitive workload scale for the different conditions is presented in Fig. 16. By definition, the single-task condition had a rating of 1.0 and the OSPAN condition had a rating of 5.0. The rating for the different IVIS interactions varied considerably across vehicles, from a low rating of 2.37 to a high of 4.57. Instances where the pairwise difference between adjacent systems was significant are denoted by an asterisk in Fig. 16.

## Discussion

The objective of the current research was to examine the effect of IVIS interactions on the cognitive workload experienced by drivers across the age range. We selected voice-based tasks that could be performed with no visual component and only a minimal button press to initiate. As such, the tasks were primarily cognitive in nature (i.e., aside from the initial button press on the steering wheel, there was no requirement for visual or manual interaction). We explored several interrelated questions concerning the cognitive workload of these voice-based tasks. First, how demanding are these IVIS interactions? How do they compare to other common in-vehicle activities such as talking on a cell phone? Does the workload differ for the different vehicles? If they differ, what is the basis for the difference? Second, laboratory studies have found that older adults exhibit greater costs when multitasking. Do these age-related differences hold for real-world interactions while operating a motor vehicle? Third, does practice eliminate any age-related or vehicle-related differences in cognitive workload? If it does, how much practice is necessary? We address each of these issues in the following paragraphs.

First, using the IVIS to complete common tasks (e.g., voice dialing, contact calling, and radio tuning) was associated with a significant increase in the cognitive workload of the driver compared to the single-task condition. The overall workload ratings associated with IVIS interaction averaged 3.34 on our 5-point scale and ranged from 2.37 to 4.57; this reflects a moderate to high level of cognitive workload. These cognitive workload ratings were associated with the intuitiveness and complexity of the IVIS and the time it took participants to complete the interaction. Systems that scored lower in cognitive workload were rated as being more intuitive and less complex and their system interactions required a shorter time to complete. By contrast, systems that were higher in cognitive workload were rated as being less intuitive and more complex and their system interactions required a greater time to complete. Importantly, our analyses were able to dissociate the differential workload associated with operating the vehicle (i.e., in the single-task condition) from the workload associated with IVIS interactions. We performed ANCOVAs that held constant single-task performance and found significant effects of the IVIS interaction. That is, the cognitive workload ratings are associated with the IVIS and not the operation of the vehicle.

**Fig. 17:**
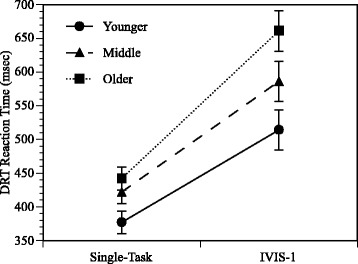
The DRT reaction time for single-task and IVIS-1 conditions after 5 days of practice. The data are plotted for younger, middle, and older age groups. *Error bars* reflect the 95 % confidence interval around the point estimate. This figure illustrates the classic age–complexity pattern, where age-related differences grow with task complexity. Moreover, it is clear that substantial costs are associated with the IVIS interactions after 5 days of practice. Hence, older adults exhibit greater costs with the IVIS interactions and practice does not eliminate the costs (for any age group)

Second, the cognitive workload experienced by older drivers performing these IVIS interactions was significantly greater than that experienced by younger drivers. This difference was revealed in the significant Condition × Age interactions, wherein performance differences between younger and older participants were amplified in the IVIS condition. For example, the age-related difference in RT in the single-task condition was 18.2 %. This age-related difference grew to 29.7 % in the on-task segments of the IVIS condition. The age-related difference in hit rates also grew from 2.1 % in the single-task condition to 8.5 % in the on-task segments of the IVIS condition.^7^ This pattern was also found in a more fine-grained analysis that was restricted to the single-task condition and on-task segments of the IVIS (i.e., IVIS-1) after 5 days of practice (Fig. 17). In this targeted analysis, there again was a Condition × Age interaction (*F*(2,266) = 12.21, *p* < 0.001, η^2^ = 0.084). An additional analysis of the log RT data also found a Condition × Age interaction (*F*(2,266) = 6.82, *p* < 0.001, η^2^ = 0.049). The age-related difference in RT in the single-task condition was 17.2 %. This age-related difference grew to 28.6 % in the on-task IVIS condition. The age-related difference in hit rate also grew from 1.7 % in the single-task condition to 11.3 % in the IVIS condition. In essence, the age-related differences that were observed in the single-task condition doubled when participants interacted with the IVIS. Older adults also rated the IVIS interactions as being more complex. These findings are in line with the *Age*–*Complexity Hypothesis* (Cerella, 1985; Cerella, Poon, & Williams, 1980) that posits that age-related differences are amplified as the complexity of the task increases. The findings are important because drivers between the ages of 55 and 64 years are the most frequent purchasers of new vehicles (Sivak, 2013). The voice-based systems found in many of these new vehicles are likely to induce high levels of cognitive workload for this cohort.

Third, practice improved performance for all conditions; however, the practice effects were greater as the task complexity increased. This was revealed in the Condition × Session interactions, where the effects of practice were more pronounced in the on-task IVIS condition than in the single-task condition. For example, RT decreased with 5 days of practice by 3.5 % in the single-task condition and by 9.0 % in the on-task segments of the IVIS condition. A similar comparison of hit rates found an increase with practice of 1.4 % in the single-task condition and of 5.7 % in the on-task IVIS condition. However, even after 5 days of practice, there were still large costs associated with IVIS interactions. A fine-grained analysis that focused on performance after 5 days of practice still found large differences between the single-task condition and on-task segments of the IVIS condition (*F*(2, 226) = 336.17, *p* < 0.001, η^2^ = 0.748). Compared to the single-task condition, RT increased by 41.8 % and hit rates decreased by 8.5 % when participants performed IVIS interactions (Fig. 17).

Practice effects for all of human learning are known to be negatively accelerated (i.e., the *Power Law of Learning*), such that the biggest improvements occur early in training (Newell & Rosenbloom, 1981; see also Heathcote, Brown, & Mewhort, 2000). This implies that any additional practice with IVIS interactions will have diminishing returns compared to what was observed after 5 days of practice. It appears that the impairments from using the IVIS cannot be easily practiced away. Moreover, neither the Condition × Session × Vehicle interactions (all *p* values ≥0.482) nor the Condition × Session × Age × Vehicle interactions (all *p* values 0.137) were significant. This is important because it indicates that the relative ordering of the IVIS systems was not altered with practice. IVIS interactions that were easy on the first day were also easy after 5 days of practice, and those IVIS interactions that were difficult on the first day were relatively difficult to perform after 5 days of practice.

### Vehicle differences

Our findings indicated that there were significant differences in the cognitive workload of the IVIS systems. The Chevy Equinox system had the lowest rating on the cognitive workload scale and the Mazda 6 system had the highest rating on the cognitive workload scale. Interestingly, the Chevy Equinox system rated highest (i.e., best) on intuitiveness, had one of the lowest ratings on complexity, and took one of the shortest time to complete (as measured by the time on task). By contrast, the Mazda 6 system rated the lowest on intuitiveness, highest on complexity, and had the second longest time to complete. This pattern is noteworthy because the intuitiveness, complexity, and time on task measures were not included in the derivation of the cognitive workload scale. Nevertheless, they converge on the same interpretation of the driver’s experience. A general principle that has emerged from this research is that robust, intuitive systems with lower levels of complexity and shorter task durations tend to have lower cognitive workload than more rigid, error-prone, time-consuming systems.

The analysis of workload using the on/off task DRT data found that “on-task” performance was associated with surprisingly high levels of workload (i.e., averaging 3.34 on our 5-point scale). The higher level of workload should serve as a caution that these voice-based interactions can be very mentally demanding and ought not to be used indiscriminately while operating a motor vehicle. It is likely that the intuitiveness, complexity, and timing demands associated with the IVIS interactions are the reason for the increased level of cognitive workload.

### Residual costs

Interestingly, the off-task DRT performance provided evidence of persistent interference following the IVIS interactions. Despite the fact that the participants were not interacting with the system in any way, there were residual costs associated with the prior interaction. These residual costs are notable for their magnitude (in the seconds immediately following an interaction, the impairments are similar to that observed with OSPAN). These costs are also notable for their duration, lasting up to 27 s after an interaction had been completed. To put this in context, at 25 mph a vehicle would have traveled 988 feet before the residual costs had completely dissipated. These findings have implications for self-regulatory strategies, such as choosing to dial or send a text message at a stoplight, because the costs of these interactions are likely to persist when the light turns green. The residual costs are likely related to the driver reestablishing situational awareness of the driving environment that was lost during the IVIS interaction (Fisher & Strayer, 2014; Strayer & Fisher, 2016).

The voice-based interactions evaluated in the current study were designed to be completed using simple voice commands. However, like others (e.g., Reimer et al., 2014), we found that many participants routinely glanced at the displays during interactions. Additionally, we found that interactions with the voice-based systems changed the frequency of glances to the forward roadway and side and rear-view mirrors. Based on these findings, it is increasingly evident that natural visual scanning behavior is fundamentally coupled to cognitive processing demands. Quite simply, it is incorrect to assume that talking to your car is an “eyes-free” activity.

## Conclusions

The current research examined the impact of IVIS interactions on the cognitive workload experienced by drivers across the age range. The data support five conclusions regarding the IVIS interactions while operating a motor vehicle.

- The momentary cognitive workload ratings associated with IVIS interaction averaged 3.34 on our 5-point scale and ranged from 2.37 to 4.57. These findings reflect a moderate to high level of cognitive workload. The workload ratings were associated with the intuitiveness and complexity of the IVIS and the time it took participants to complete the interaction.
- The momentary cognitive workload experienced by older drivers performing the IVIS interactions was significantly greater than that experienced by younger drivers. In fact, the age-related differences that were observed in the single-task condition doubled when participants interacted with the IVIS. Practice does not eliminate the interference caused by IVIS interactions. IVIS interactions that were easy on the first day were also easy after 5 days of practice and those interactions that were difficult on the first day were still relatively difficult to perform after 5 days of practice.
- There were differences in the cognitive workload of the different IVIS systems over and above any differences associated with simply driving the vehicles. We found that robust, intuitive systems with lower levels of complexity and shorter task durations tend to have lower cognitive workload than more rigid, error-prone, time-consuming systems.
- There were long-lasting residual costs after IVIS interactions had terminated. At 3 s after the IVIS interaction had completed, the cognitive workload was a category 5 level; at 6 s the workload was at a category 4 level; at 9 s the workload was at a category 3 level; at 15 s the workload was at a category 2 level; and the workload reached a category 1 level after 27 s.

## Footnotes

1 The OSPAN task was originally developed to measure working memory capacity (e.g., Engle, 2012). An auditory version of the OSPAN task was developed for use in the vehicle by Watson and Strayer (2010).

2 The analyses reported below show the same pattern as when age is treated as a continuous variable rather than a categorical variable.

3 The Vehicle condition codes for all data collected in each vehicle. Thus, a significant effect of Vehicle would reflect general differences in performance associated with *driving* the vehicle and not differences in the IVIS interface. Differences in the IVIS interfaces are seen in the effect of Condition and the Condition × Vehicle interaction.

4 A preliminary analysis that included gender as a factor found that males responded, on average, 45 ms faster than females (*p* < 0.001); however, gender did not interact with any of the other factors (all *p* values >0.200); hence we collapsed across this variable for all additional analyses.

5 Interpretation of the Condition × Age interaction is complicated by the possibility that age-related differences in dual-task performance may simply reflect an increase that is proportional to the age-related slowing observed in the single-task baseline (e.g., Lindholm & Parkinson, 1983). However, a log transformation of the RT data makes proportional changes additive, whereas an interaction would provide evidence that the dual-task costs were greater for older adults (Madden, 2001). The significant Condition × Age interaction found in the log-transformed RT data indicates that the dual-task costs experienced by older adults are greater than would be expected by the proportional increase.

6 The z-transformation is a linear transformation whereby the transformed mean is 0 and the standard deviation is 1. The resulting data are centered and differences between the conditions are proportional to the differences before the transformation (i.e., an ANOVA performed on the z-transformed data is identical to an analysis on the raw data).

7 These data rule out speed–accuracy tradeoffs as an explanation of the age-related differences in IVIS interaction. For both RT and accuracy measures, older adults’ performance was impaired to a greater extent than that of younger adults.

## References

[CR1] Bergen B, Medeiros-Ward N, Wheeler K, Drews F, Strayer DL (2013). The crosstalk hypothesis: Language interferes with driving because of modality-specific mental simulation. Journal of Experimental Psychology: General.

[CR2] Carney C, McGehee D, Harland K, Weiss M, Raby M (2015). Using naturalistic driving data to access the prevalence of environmental factors and driver behaviors in teen driver crashes.

[CR3] Cerella J (1985). Information processing rates in the elderly. Psychological Bulletin.

[CR4] Cerella J, Poon LW, Williams DM, Poon LW, Poon LW (1980). Age and the complexity hypothesis. Aging in the 1980s: Psychological issues.

[CR5] Cooper, J. M., Ingebretsen, H., & Strayer, D. L. (2014). *Measuring Cognitive Distraction in the Automobile IIa: Mental Demands of Voice-Based Vehicle Interactions with OEM Systems*. Washington, DC: AAA Foundation for Traffic Safety. Retrieved from https://www.aaafoundation.org/sites/default/files/Cog%20Distraction%20Phase%20IIA%20FINAL%20FTS%20FORMAT.pdf.

[CR6] Engle RW (2012). Working memory capacity as executive attention. Current Directions in Psychological Science.

[CR7] Fisher DL, Strayer DL (2014). Modeling situation awareness and crash risk. Annals of Advances in Automotive Medicine.

[CR8] Hart, S. G., & Staveland, L. E. (1988). Development of NASA-TLX (Task Load Index): Results of empirical and theoretical research. In P. A. Hancock & N. Meshkati (Eds.), *Human mental workload*. Amsterdam: North Holland Press. 139–183.

[CR9] Hartley AA, Little DM (1999). Age-related differences and similarities in dual task interference. Journal of Experimental Psychology: General.

[CR10] Heathcote A, Brown S, Mewhort DJK (2000). The power law repealed: The case for an exponential law of practice. Psychonomic Bulletin & Review.

[CR11] ISO DIS 17488 (2015). *Road Vehicles--Transport information and control systems--Detection Response Task (DRT) for assessing selective attention in driving*. Draft International Standard, ISO TC 22/SC39/WG8.

[CR12] Kelley JF (1983). An empirical methodology for writing user-friendly natural language computer applications. Proceedings of ACM SIG-CHI ’83 human factors in computing systems.

[CR13] Kramer AF, Larish J, Rogers W, Fisk AD, Walker N (1996). Aging and dual-task performance. Aging and skilled performance.

[CR14] Lee JD, Caven B, Haake S, Brown TL (2001). Speech-based interactions with in-vehicle computers: The effect of speech-based e-mail on drivers’ attention and roadway. Human Factors.

[CR15] Lindholm, J. M., & Parkinson, S. R. (1983). An interpretation of age-related differences in letter matching performance. *Perception & Psychophysics, 33*, 283–294. 10.3758/bf03202866 6866689

[CR16] Madden DJ, Birren JE, Schaie KW (2001). Speed and timing of behavioral processes. Handbook of the psychology of aging.

[CR17] McDowd JM, Shaw RJ, Craik FIM, Salthouse TA (2000). Attention and aging: A functional perspective. The handbook of aging and cognition.

[CR18] National Safety Council White Paper (2010). Understanding the distracted brain: Why driving while using hands-free cell phones is risky behavior. Retrieved from https://www.fnal.gov/pub/traffic_safety/files/NSC%20White%20Paper%20-%20Distracted%20Driving%203-10.pdf

[CR19] Newell A, Rosenbloom PS, Anderson JR (1981). Mechanisms of skill acquisition and the law of practice. Cognitive skills and their acquisition.

[CR20] Reimer, B., Mehler, B., Dobres, J., McAnulty, H., Mehler, A., Munger, D., & Rumpold, A. (2014). Effects of an ‘expert mode’ voice command system on task performance, glance behavior & driver physiology. *Proceedings of the 6th International Conference on Automotive User Interfaces and Interactive Vehicle Applications (AutoUI 2014)*. Seattle.

[CR21] Shiffrin RM, Schneider W (1977). Controlled and automatic human information processing: II. Perceptual learning, automatic attending, and a general theory. Psychological Review.

[CR22] Sivak, M. (2013). Marketing implications of the changing age composition of vehicle buyers in the U.S. Online publication downloaded on August 3, 2015 from http://deepblue.lib.umich.edu/bitstream/handle/2027.42/97760/102946.pdf?sequence=1&isAllowed=y

[CR23] Strayer DL, Cooper JM, Turrill J, Coleman JR, Hopman RJ (2015). The smartphone and the driver’s cognitive workload: A comparison of Apple, Google, and Microsoft’s intelligent personal assistants.

[CR24] Strayer DL, Fisher DL (2016). SPIDER: A model of driver distraction and situation awareness. Human Factors.

[CR25] Strayer DL, Turrill J, Coleman J, Ortiz E, Cooper JM (2014). Measuring cognitive distraction in the automobile: II. Assessing in-vehicle voice-based interactive technologies.

[CR26] Strayer DL, Turrill J, Cooper JM, Coleman J, Medeiros-Ward N, Biondi F (2015). Assessing cognitive distraction in the automobile. Human Factors.

[CR27] Watson JM, Strayer DL (2010). Supertaskers: Profiles in extraordinary multi-tasking ability. Psychonomic Bulletin and Review.

